# An evaluation of the long-term effectiveness of Gatekeeper™ intersphincteric implants for passive faecal incontinence

**DOI:** 10.1007/s10151-022-02630-z

**Published:** 2022-05-20

**Authors:** S. A. A. Jabbar, J. Camilleri-Brennan

**Affiliations:** 1 Department of Colorectal Surgery, Forth Valley Royal Hospital, Larbert, Stirlingshire Scotland, UK; 2 Department of Surgery, University of Glasgow, Glasgow, Scotland, UK

**Keywords:** Faecal incontinence, Gatekeeper™, Bulking agents, Artificial anal sphincter, Endoanal ultrasonography

## Abstract

**Background:**

Implantation of Gatekeeper™ prostheses presents an option for the treatment of passive faecal incontinence (FI). Whilst preliminary results are encouraging, long-term data regarding its sustained benefit are limited. The aim of this study was to assess and evaluate the long-term clinical function and quality of life of patients with passive faecal incontinence who were treated with Gatekeeper™ prostheses.

**Methods:**

This was a single centre, single surgeon retrospective study of prospectively collected clinical data in patients with FI treated between June 2012 and May 2019. Patients with passive FI with symptoms refractory to conservative treatment and endoanal ultrasonography showing intact or disrupted internal anal sphincter were included. Formal clinical and quality of life assessments were carried out using the St. Mark’s Incontinence Score (SMIS) and Faecal Incontinence Quality of Life (FIQoL) questionnaires at baseline, 3 months, 6 months, 12 months and then annually. Endoanal ultrasonography was performed both before and after surgery.

**Results:**

Forty patients (14 males, 26 females) with a median age of 62.5 (range 33–80) years were treated with the Gatekeeper™ implant. The majority of patients (87.5%) received six implants. There were no peri or post-operative complications. Prosthesis migration was observed in 12.5% patients. The median follow-up duration was 5 years (interquartile range (IQR) 3.25–6.00 years). A sustained improvement in median SMIS and FIQoL scores from baseline to follow-up was noted. Significant differences were observed between the median baseline SMIS score and last follow-up score of 16.00 (IQR 15.00–16.75) to 7.00 (IQR 5.00–8.00) respectively (*p* < 0.001), a 56.25% decrease. The overall median FIQoL score showed a significant improvement from 7.95 (IQR 7.13–9.48) to 13.15 (IQR 12.00–13.98) (*p* < 0.001) a 65.40% increase.

**Conclusions:**

Gatekeeper™ implantation is a safe approach to treating passive FI and is minimally invasive, reproducible and has minimal complications. Long-term sustained clinical improvement is achievable beyond 5 years. Careful patient selection is paramount, as is consistency of technique and follow-up protocol.

## Introduction

Faecal incontinence (FI), the involuntary loss of solid or liquid stool, has a significant impact on the psychological wellbeing and quality of life (QoL) of patients [1]. The prevalence rate can be as high as 20% in the adult population and rises exponentially with age [2, 3]. Due to the multifactorial aetiology of FI, its management is both complex and multidisciplinary [4, 5]. First line management, as recommended by the UK National Institute for Clinical Excellence (NICE) guidelines, should be conservative, including patient education, dietary advice, bowel habit modification, anti-diarrhoeal medication and pelvic floor exercises [6]. Alternative non-surgical therapeutic options include biofeedback and rehabilitation; however, the results of these modalities are variable [7–9]. In cases refractory to conservative approaches surgical options, which include the use of anal bulking agents, are available. These bulking agents are considered to work by augmenting the size of the anal sphincter and increasing the pressure within [10]. The indications for the use of bulking agents vary from study to study and include: (1) failed conservative measures; (2) intact but weak internal anal sphincter (IAS); (3) IAS damage (childbirth, haemorrhoidectomy, anal dilation, sphincterotomy); (4) External anal sphincter (EAS) defect. The Gatekeeper™ (GK; THD SpA, Correggio, Italy) procedure, introduced in 2011, involves injection of 4–6 self-expanding polyacrylonitrile (Hyexpan) implants into the intersphincteric space [11]. Preliminary results have been encouraging however there is limited long-term follow-up beyond 5 years to show sustained benefits of such bulking agents [11, 12]. The primary aim of this study was to assess and evaluate the long-term clinical function and quality of life of patients with passive FI who were treated with Gatekeeper™ prostheses. The secondary aim was the assessment of post-operative complications such as surgical site infection and prosthesis displacement.

## Materials and methods

This is a single centre, single surgeon retrospective analysis of prospectively collected clinical data in patients with FI treated between June 2012 and May 2019 at Forth Valley Royal Hospital (Stirlingshire, Scotland, United Kingdom). Informed written consent was obtained from all patients included in the study. Data were stored on a secure encrypted electronic database.

### Patient selection and assessment

Patient selection was based on data from history taking, including cognitive assessment, and physical examination of each patient. History of anorectal and gynaecological surgery was noted. FI was defined as incontinence to gas, liquid and/or solid stool more than once weekly with onset within at least 6 months before recruitment. Characteristics of faecal consistency and frequency of FI were recorded. Formal clinical and QoL assessments were carried out using the validated St. Mark’s Incontinence Score (SMIS) [13], sometimes referred to as Vaizey score, which ranges from 0 (fully continent) to 24 (complete incontinence), and Faecal Incontinence Quality of Life (FIQoL) [14] questionnaires, whereby the higher the score the better the QoL. A colonoscopy was performed to exclude colorectal disease. Endoanal ultrasonography (EAUS) was performed to assess anorectal morphology and to diagnose and evaluate the extent of any sphincter lesions.

Inclusion criteria for the study were: (1) patients over the age of 18 with passive FI for at least 6 months (idiopathic or after anal surgery); (2) symptoms refractory to at least 6 months of conservative treatment (according to the NICE criteria this includes pelvic floor physiotherapy, dietary modification, anal plugs and anti-diarrhoeal medication) and (3) EAUS showing normal, degenerated or disrupted internal anal sphincters (IAS) only (Sphincter degeneration is noted on EAUS as an internal anal sphincter that is thin, hypoechogenic and has a poorly defined edge, in the absence of structural damage). Exclusion criteria were: (1) urge faecal incontinence; (2) EAUS proven external anal sphincter defect; (3) active perianal infection; (4) inflammatory bowel disease with perianal involvement; (5) altered cognitive status preventing compliance; (6) anorectal malignancy;(7) previous pelvic radiotherapy; (8) previous anorectal surgery for congenital malformations.

### Operative technique

The operative technique has been described elsewhere [15]. Using a custom THD Gatekeeper™ Delivery System (THD SpA, Correggio, Italy) and dispenser the GK prostheses are placed in the intersphincteric space of the mid to upper anal canal. The dehydrated GK prostheses are 22 mm long and 2 mm in diameter. Due to their hydrophilic properties, the GK prostheses expand and become shorter and thicker 48 h following implantation in human tissue, increasing from a volume of 70 to 500 mm^3^ [11]. The procedure is carried out under general or regional anaesthesia. Patients are given a phosphate enema preoperatively. Intravenous gentamicin 1.5 mg/kg and metronidazole 500 mg antibiotics are given at anaesthetic induction. The patient is prepped with betadine and draped creating a sterile field. Using an anal retractor, the IAS and intersphincteric groove are defined. A small 2 mm skin incision is made 2 cm from the anal verge in the perianal area. Once the dispenser is connected to the delivery system, the needle is inserted through the skin incision and guided into the intersphincteric space and tunnelled with the tip lying just beyond the dentate line. Satisfactory needle position is confirmed by digital palpation and/or endoanal ultrasound and the gun is fired causing cannula retraction and prosthesis implantation into the intersphincteric space. These steps are repeated with incisions equidistant from each other and 4–6 GK implants are inserted. The prostheses are designed to self-fix into position to reduce risk of migration. The skin wounds at the end of the procedure are closed with absorbable sutures. Oral metronidazole 400 mg three times daily is prescribed for a 5 day course post-operatively and laxatives are also given to prevent constipation. Advice is given to avoid any anal trauma for at least 72 h postoperatively.

### Follow-up

A follow-up protocol was set for all patients. First postoperative evaluation was carried out at 6 weeks which included SMIS and FIQoL questionnaires and EAUS to assess implant position. Thereafter faecal continence status was assessed using SMIS and FIQoL questionnaires in the clinic or via a telephone consultation at 3 months, 6 months, 12 months and then annually. Last follow-up was recorded as the most recent FI evaluation at the time of data analysis for the study. Improvement of FI was classified as a 50% improvement in scores from baseline as similarly described in other studies [16].

### Statistical analysis

A descriptive analysis of patients’ characteristics, symptom scores and EAUS findings was carried out. Quantitative data is presented as median and interquartile range (IQR). To compare the baseline versus the last follow-up SMIS and FIQoL data, the Wilcoxon signed-rank test was used and *p* < 0·05 was considered statistically significant. SPSS version 26.0 (IBM, Armonk, N Y, USA) was used for statistical analysis.

## Results

In the study period between June 2012 and May 2019, 40 patients (14 males, 26 females) with a median age of 62.5 years (range 33 ò–80 years) were treated for FI with GK implants. Table 1 summarises the demographic and the characteristics data of the patients included in the study. Baseline EAUS showed an intact IAS in 20 patients but there was IAS degeneration in 18 patients. Two patients had partial thickness defects in the IAS which involved less than one quadrant.

**Table 1:** Demographic and characteristics data of the patients included in the study (*n*=40)

Characteristic	Value
Age (years)	62.5 (33–80)^a^
Sex M/F	14 (35%)/26 (65%)
Previous anorectal and gynaecological surgery	
Haemorrhoidectomy	4
Anal stretch	1
Stapled anopexy	1
Internal anal sphincterotomy	2
Sacral nerve stimulation	1
Pelvic floor repair	1
Colporrhaphy	2
Pre-op EAUS	
Sphincter defect	2 (5%)
IAS	1
EAS	0
IAS + IAS degeneration	1
IAS degeneration	18 (45%)
No of implants	
4 implants	3
5 implants	2
6 implants	35

*IAS* internal anal sphincter, *EAS* external anal sphincter

^a^Median (range)

The majority of patients (87.5%) received 6 GK implants as described in the original procedure. No intraoperative, immediate or early postoperative complications occurred.

The median follow-up duration was 5 years (IQR 3.25 6 years).Three patients died during the follow-up period. On initial postoperative imaging all prostheses achieved the correct size, however, 2 patients were noted to have 1 partial prosthetic migration and 3 patients had between 1 to 3 non-detectable implants suggestive of complete prosthetic migration. Re-implantation was performed for 2 patients at 2 years following their primary procedure.

**Fig. 1:**
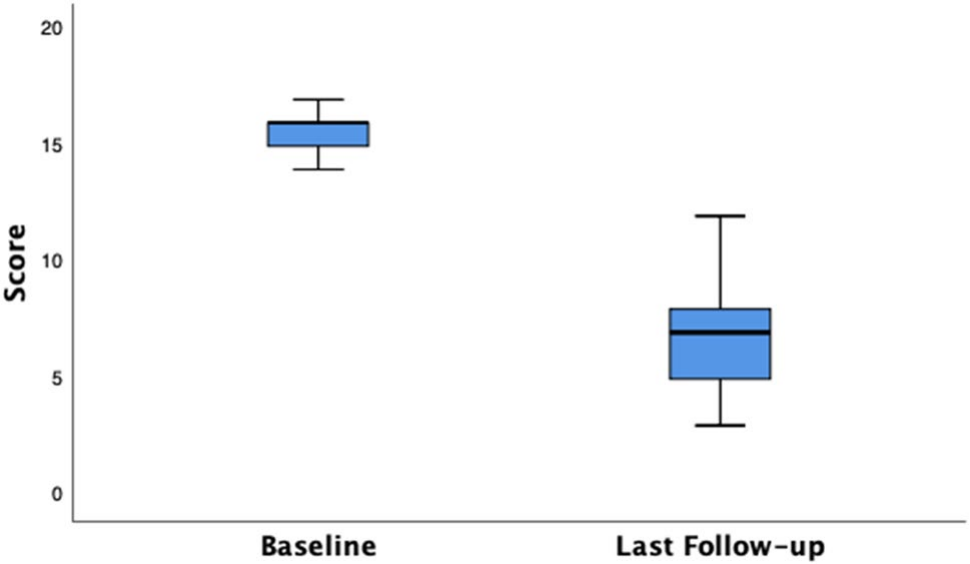
Boxplot of SMIS—Baseline and Last Follow-up. Baseline median SMIS, 16.00 (IQR 15.00–16.75) versus last follow-up median score, 7.00 (IQR 5.00–8.00)

**Fig. 2:**
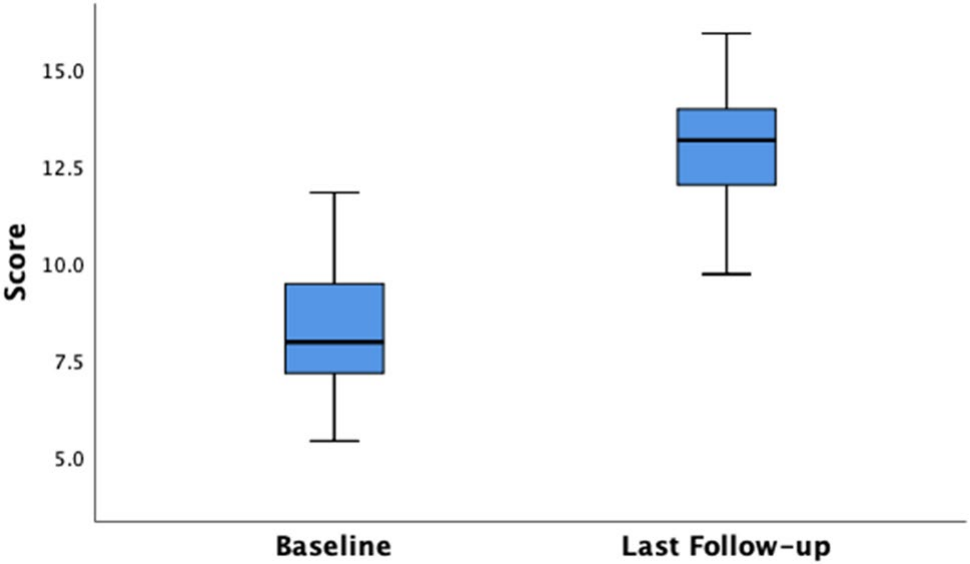
Boxplot of Total FIQoL Scores—Baseline and Last Follow-up. Baseline median FIQoL Score, 7.95 (IQR 7.13–9.48) versus last follow-up median score, 13.15 (IQR 12.00–13.98)

The clinical outcomes are shown in Table 2. A sustained improvement in median SMIS scores is shown from baseline to throughout the follow-up period. A similar sustained postoperative improvement is also noted within each of the quality of life domains in the FIQoL scores from baseline to follow-up (Table 3). Significant differences can be observed between the median baseline SMIS score and last follow-up score of 16.00 (IQR 15.00–16.75) to 7.00 (IQR 5.00–8.00) respectively (*p* < 0.001) (Fig. 1). With regard to the FIQoL, baseline median scores of lifestyle 1.80 (IQR 1.60–2.08), coping 2.00 (IQR 1.73–2.08), depression 2.55 (IQR 2.20–3.00) and embarrassment 1.70 (IQR 1.30–2.00) showed a significant improvement compared to final follow-up scores of each domain, 3.00 (IQR 3.00–3.38), 3.10 (IQR 3.00–3.50), 3.50 (IQR 3.23–3.95), 3.15 (IQR 3.00–3.30), respectively. Overall FIQoL scores significantly improved from 7.95 (IQR 7.13–9.48) to 13.15 (IQR 12.00–13.98) (*p* < 0.001). Figures 1 and 2 illustrate overall improvements in median and interquartile SMIS and FIQoL scores.

**Table 2:** SMIS at baseline (preop) and during postoperative follow-up intervals

	Pre-op (*n* = 40)	6 weeks (*n* = 40)	3 months (*n* = 40)	6 months (*n* = 40)	1 year (*n* = 40)	2 years (*n* = 39)	3 years (*n* = 37)	4 years (*n* = 30)	5 years (*n* = 24)	6 years (*n* = 17)	7 years (*n* = 9)	8 years (*n* = 3)
SMIS	16.00 (15.00–16.75)	6.00 (5.00–7.75)	5.00 (4.00–7.00)	5.00 (4.00–7.00)	5.00 (4.00–7.00)	5.00 (4.00–7.00)	5.00 (4.00–7.00)	5.00 (4.75–7.25)	6.50 (5.00–9.00)	7.00 (5.00–8.00)	6.00 (4.50–6.50)	5.00 (3.00–5.00)

Values are presented as median (IQR)

*SMIS* St. Mark’s Incontinence Score

**Table 3:** FIQoL scores at baseline (preop) and during postoperative follow-op intervals

	Preop (*n* = 40)	6 months (*n* = 40)	1 year (*n* = 40)	2 years (*n* = 39)	3 years (*n* = 38)	4 years (*n* = 30)	5 years (*n* = 24)	6 years (*n* = 18)	7 years (*n* = 8)	8 years (*n* = 3)
FIQoL total	7.95 (7.12–9.48)	13.05 (12.10–13.98)	13.15 (12.23–13.88)	13.10 (12.10–13.90)	13.10 (12.10–14.00)	13.10 (12.30–14.10)	12.85 (12.08–14.00)	13.60 (12.45–14.18)	13.00 (12.25–13.78	13.20 (12.80–13.20)
FIQoL lifestyle	1.80 (1.60–2.08)	3.00 (3.00–3.38)	3.20 (3.00–3.40)	3.00 (3.00–3.30)	3.00 (3.00–3.33)	3.10 (3.00–3.40)	3.10 (3.00–3.38)	3.25 (3.00–3.43)	3.00 (2.93–3.20)	3.20 (3.00–3.20)
FIQoL coping/behaviour	2.00 (1.72–2.08)	3.20 (3.00–50)	3.15 (3.00–3.50)	3.20 (3.00–3.50)	3.15 (3.0–3.53)	3.30 (3.00–3.60)	3.25 (3.00–3.50)	3.40 (3.00–3.63)	3.35 (3.03–3.68)	3.20 (3.00–3.20)
FIQoL depression	2.55 (2.20–3.00)	3.55 (3.33–3.95)	3.50 (3.23–3.95)	3.50 (3.20–3.80)	3.50 (3.20–4.00)	3.55 (3.40–3.55)	3.50 (3.33–3.95)	3.50 (3.38–3.85)	3.45 (3.10–3.80)	3.50 (3.50–3.50)
FIQoL embarrassment	1.70 (1.30–2.00)	3.00 (3.00–3.30)	3.15 (3.00–3.30)	3.30 (3.00–3.30)	3.30 (3.00–3.30)	3.30 (3.00–3.40)	3.30 (3.00–3.30)	3.30 (3.00–3.70)	3.30 (3.00–3.30)	3.30 (3.00–3.30)

Values are presented as median (IQR)

*FIQoL* Faecal Incontinence Quality of Life

## Discussion

This study was an analysis of the long-term outcomes following the implantation of the GK prostheses in patients with passive FI. Strict inclusion criteria were adhered to, rather than accepting all-comers with any type of FI. The focus was to investigate the outcomes of the use of the GK in a more defined group to highlight the value of careful patient selection and also provide a longer follow-up period than what has previously been reported in the literature. The majority of patients (95%) had an intact IAS with 18 (45%) patients having an attenuated IAS suggestive of degeneration. Many studies have reported use of bulking agents for patients with some EAS dysfunction or defect [11, 12, 17–20]. Due to the multifactorial nature of FI, patients often have mixed urge and passive incontinence. This can make interpretation of results difficult. In this study, the focused indication of passive FI only was used. This is supported by other studies which avoided heterogenous selection [21–24].

Effective therapeutic options for passive FI are scarce [25]. Since the pioneering efforts of Shafik who used autologous fat, within the last 20 years there have been a number of papers describing the results of injectable agents for FI such as collagen (Permacol) and silicone (PTQ or Bioplastique). The results of these studies have, however, been very variable and inconsistent [26]. One of the major issues with these implants was the reduced efficacy due to degradation, resorption or migration of the injected material [27]. Equally, there is limited long-term evidence over 2 years to show durability of previously used injectable bulking agents.

GK, however, has been shown to be an effective treatment modality. The first experience reported was by Ratto et al. 2011 who showed a clinically significant improvement in FI in 13 patients and sustained improvement in Wexner, Vaizey, Health Survey (SF36) and FIQoL scores with median follow-up of 12 months [11]. A comparative retrospective study carried out by Parello et al. in 2012 compared 7 GK patients to 6 sacral nerve stimulation patients and showed a sustained improvement in Wexner continence scores with a median follow-up of 18 months for GK and 20 months for SNS cases [28]. Fabiani et al. used GK and showed a Wexner continence score less than 4 for 6 patients at 6 months average follow-up. Biondo et al. reported GK implantation as being safe and effective and showed significant differences between baseline mean Vaizey scores at 6 months, 12 months and last follow-up [29]. Long term follow-up in this study was 2.7 years. In a multicentre study of 54 patients with follow-up of 1 year, Ratto et al. noted that after GK implantation, FI severity scores reduced and patients’ quality of life increased [12, 29]. These studies all report that GK implants are safe and effective and our study would support these findings. In our study GK was found to be safe with no peri or post operative complications other than prothesis migration in a few patients. With regard to outcomes there was a sustained improvement in median SMIS and FIQoL scores from baseline to follow-up.

Since its introduction, studies have shown GK to be safe and effective. However, to our knowledge, there is currently no published data on the long-term clinical outcomes of GK usage. The median follow-up duration of our study was 5 years (IQR 3.25 6 years) and, as discussed earlier, sustained efficacy of GK implantation was shown both with the SMIS and FIQoL scores.

Our data also demonstrates the safety of the GK implants. There were no episodes of acute or chronic sepsis at the implantation sites. None of the patients reported anorectal pain or obstructed defaecation.

The mechanism of action of the GK prostheses has been the subject of some debate. It is thought that implantation into the intersphincteric space plays a key role as it bulks up the size of the anal sphincter, the result being an improved seal of the anal canal at rest and potential improvement of anal resting pressure [11]. In the context of an IAS defect, an implant placed adjacent to the defect provides symmetry and improved configuration of the anal canal [30]. In a recent study from Ratto’s group, Grossi et al. suggests that ‘morphofunctional changes in EAS may lead to improved squeeze function after GK implant’ [31]. This function of GK may therefore be of potential benefit to patients with urge and mixed incontinence, rather than passive incontinence.

Crucially, the intersphincteric location of implant deployment potentially prevents migration or extrusion in comparison to deployment in the submucosal space. Implantation in the mid to upper anal canal is also vital. The quick expansion of GK after deployment also theoretically reduces risk of migration. In this study, 2 patients were observed to have 1 partial prosthetic migration and 3 patients had between 1 and 3 non-detectable implants suggestive of complete prosthetic migration (12.5% patients). Re-implantation was performed for 2 patients at 2 years following their primary procedure with no difference in sustained long-term efficacy in comparison to other patients. Studies report frequent prosthesis migration and it has been suggested that incorrect positioning, the virtual space and stretching during defecation can all contribute to implant displacement [32]. Our rate of prosthesis migration was indeed lower than that observed in the literature which reportedly range between 14 and 71% [12, 32]. Interestingly, it should be considered that the displacement of prostheses is potentially the main cause for decline in therapeutic effect [21, 27, 32, 33]. The low prostheses displacement rate observed in this study could explain the sustained long-term efficacy beyond 5 years, although a further EAUS assessment would give a more accurate evaluation of this observation.

This study has a number of limitations, one of which is the relatively small sample size. Another limitation of this study is that not all patients completed their 5 year follow-up. However, the patients that completed the assessments at 5 or more years showed sustained long-term clinical improvement. Based on these results, it is expected that the other patients would have similar outcomes. Since this study is based on the experience of a single colorectal unit, the results may not necessarily translate to other centres.

While this study was being performed the GK was further developed into the SphinKeeper™ (SK) [34]. The SK differs from the GK in terms of the increased size and number of prostheses, the arrangement of these prostheses being not dissimilar to an artificial anal sphincter. The properties of the SK could potentially allow it to be employed in patients with FI of diverse aetiologies, potentially treating patients with larger anal sphincter defects, in view of the improved sphincter contractility with SK when compared to GK [35]. Given the improved long-term sustained results in our patients we anticipate that patients who undergo the SK procedure will have similarly good outcomes. However, this will need to be assessed in further studies.

## Conclusions

The key findings of this study are that the GK implant is a safe approach to managing passive FI and can provide long term sustained improvement in patient symptoms. We have demonstrated the advantages of the procedure in that it is minimally invasive, reproducible and has minimal complications. The results were maintained over time and, as demonstrated in this study, efficacy can last beyond 5 years.

## Data Availability

The datasets analysed during the current study are available from the corresponding author on reasonable request.
