# Correction to: An evaluation of the long‑term effectiveness of Gatekeeper™ intersphincteric implants for passive faecal incontinence

**DOI:** 10.1007/s10151-022-02655-4

**Published:** 2022-06-22

**Authors:** S. A. A. Jabbar, J. Camilleri-Brennan

**Affiliations:** 1 Department of Colorectal Surgery, Forth Valley Royal Hospital, Larbert, Stirlingshire Scotland, UK; 2 Department of Surgery, University of Glasgow, Glasgow, Scotland, UK

## Correction to: Techniques in Coloproctology (2022) 26:537–543 10.1007/s10151-022-02630-z

The article An evaluation of the long‑term effectiveness of Gatekeeper™ intersphincteric implants for passive faecal incontinence, written by S. A. A. Jabbar, J. Camilleri‑Brennan, was originally published Online First without Open Access. After publication in volume 26, issue 7, pages 537–543 the author decided to opt for Open Choice and to make the article an Open Access publication. Therefore, the copyright of the article has been changed to © The Author(s), 2022 and the article is forthwith distributed under the terms of the Creative Commons Attribution 4.0 International License, which permits use, sharing, adaptation, distribution and reproduction in any medium or format, as long as you give appropriate credit to the original author(s) and the source, provide a link to the Creative Commons licence, and indicate if changes were made. The images or other third party material in this article are included in the article’s Creative Commons licence, unless indicated otherwise in a credit line to the material. If material is not included in the article’s Creative Commons licence and your intended use is not permitted by statutory regulation or exceeds the permitted use, you will need to obtain permission directly from the copyright holder. To view a copy of this licence, visit http://creativecommons.org/licenses/by/4.0/.

The original article has been corrected.

